# Artificial intelligence-based education assists medical students’ interpretation of hip fracture

**DOI:** 10.1186/s13244-020-00932-0

**Published:** 2020-11-23

**Authors:** Chi-Tung Cheng, Chih-Chi Chen, Chih-Yuan Fu, Chung-Hsien Chaou, Yu-Tung Wu, Chih-Po Hsu, Chih-Chen Chang, I-Fang Chung, Chi-Hsun Hsieh, Ming-Ju Hsieh, Chien-Hung Liao

**Affiliations:** 1 Department of Traumatology and Emergency Surgery, Chang Gung Memorial Hospital, Chang Gung University, 5 Fu-Hsing Street, Kwei-Shan District, Taoyuan, Taiwan; 2 Center for Artificial Intelligence in Medicine, Chang Gung Memorial Hospital, Linkou, Taoyuan, Taiwan; 3 Institute of Biomedical Informatics, National Yang-Ming University, Taipei, Taiwan; 4 Department of Rehabilitation and Physical Medicine, Chang Gung Memorial Hospital, Chang Gung University, Linkou, Taoyuan, Taiwan; 5 Department of Emergency Medicine, Chang Gung Memorial Hospital, Chang Gung University, Taoyuan, Taiwan; 6 Medical Education Research Center, Chang Gung Memorial Hospital, Chang Gung University, Taoyuan, Taiwan; 7 Department of Medical Imaging and Intervention, Chang Gung Memorial Hospital, Chang Gung University, Taoyuan, Linkou, Taiwan; 8 School of Medicine, Chang Gung University, Taoyuan, Taiwan

**Keywords:** Medical image education, Artificial intelligence, Deep learning, Fracture, Personalized education

## Abstract

**Background:**

With recent transformations in medical education, the integration of technology to improve medical students’ abilities has become feasible. Artificial intelligence (AI) has impacted several aspects of healthcare. However, few studies have focused on medical education. We performed an AI-assisted education study and confirmed that AI can accelerate trainees’ medical image learning.

**Materials:**

We developed an AI-based medical image learning system to highlight hip fracture on a plain pelvic film. Thirty medical students were divided into a conventional (CL) group and an AI-assisted learning (AIL) group. In the CL group, the participants received a prelearning test and a postlearning test. In the AIL group, the participants received another test with AI-assisted education before the postlearning test. Then, we analyzed changes in diagnostic accuracy.

**Results:**

The prelearning performance was comparable in both groups. In the CL group, postlearning accuracy (78.66 ± 14.53) was higher than prelearning accuracy (75.86 ± 11.36) with no significant difference (*p* = .264). The AIL group showed remarkable improvement. The WithAI score (88.87 ± 5.51) was significantly higher than the prelearning score (75.73 ± 10.58, *p* < 0.01). Moreover, the postlearning score (84.93 ± 14.53) was better than the prelearning score (*p* < 0.01). The increase in accuracy was significantly higher in the AIL group than in the CL group.

**Conclusion:**

The study demonstrated the viability of AI for augmenting medical education. Integrating AI into medical education requires dynamic collaboration from research, clinical, and educational perspectives.

## Key points

- A heatmap-producing radiography reading system can be utilized in medical education.
- Artificial intelligence (AI) can offer low-level supervision for medical students to read hip fracture images.
- AI proved viable for augmenting and being integrated into medical education.

## Introduction

The history of medical education reform amply demonstrates that curricular change has been incremental, reactive, and mostly marginalized [1, 2]. With recent transformations in medical education, many efforts have been made to integrate technology and ethical aspects to efficiently improve the professionalism and clinical ability of medical students and trainees [3, 4]. Medical image learning is experience dependent, and trainees need to learn several kinds of images to achieve an expert level. This training requires acquiring skills to analyze and extract imaging features to identify patterns, generate a differential diagnosis that matches the patterns, and correlate the imaging features and differential with clinical findings to select the most likely diagnosis [5]. However, owing to time constraints, training opportunities might be compressed, and trainees may not be able to access as many images as their tutors or teachers. Individual variability is thought to substantially affect learning styles [6, 7]. Moreover, learning is dictated by the number and diversity of cases encountered, with varying practices and patient mixes. An artificial intelligence (AI)-assisted teaching platform can deliver personalized education and 24-h supervised tutoring that benefits both trainees and trainers. AI generated from deep neural network learning has been developed to help deliver precision medicine and health services [3, 8]. Researchers are increasingly embracing this modality to develop tools for diagnosis and prediction as well as to improve the effectiveness of healthcare. However, AI applications to medical education have been relatively underexplored. Although a tremendous amount of research has focused on AI in decision support [9–13], very little has focused on personalized medical education. Additionally, several AI methods use e-learning modules to identify the learning styles of individual students [6, 14, 15].

In this study, we developed a prospective blind image education system and confirmed that AI can support medical students and help them learn with confidence.

## Materials and methods

### Study population

This research involved the participation of students from the Medical School at Chang Gung University, Taiwan. We recruited undergraduate students in their fifth year of medical school training who had finished their basic medicine classes and entered the clinical rotation. All the participants volunteered to join this project. The study was designed as a single-center randomized controlled trial (RCT) conducted in the medical faculty of Chang Gung Memorial Hospital, Taiwan, between January 2020 and July 2020. At the beginning of the study, we introduced the study design, the method of image collection, the reading method of pelvis anteroposterior (AP) view radiographs (PXRs), and the characteristic features of hip fractures. The participants were enrolled in the study after an informed consent process.

### The AI education system—HipGuide

We collected 3605 PXRs, which are commonly used to diagnose hip fractures, from the patients recorded in the trauma registry of January 2008 to December 2016 to train a hip fracture detection AI system, “HipGuide,” using a deep learning algorithm of DenseNet-121. All the images were reviewed by a trauma specialist with clinical information including injury mechanism, advanced images, surgical reports, and final diagnosis in the medical record. The development dataset contains 1975 (54.8%) images with hip fracture (including femoral neck fracture and trochanteric fracture) and 1630 (45.2%) images without hip fracture. The technical part of the system development is detailed in our previous work [16]. After the model trained, we collected an independent test set using 50 hip fracture and 50 normal PXR from 2017 for validation. When tested on new test images, the algorithm generates a probability of hip fracture, and an overlay heatmap representing the area contributes to this decision using a grad-CAM algorithm. The AI system achieved 91% accuracy and 98% sensitivity in the independent test set, and the heatmap identified 95.9% of the fracture sites. The model and parameters were adjusted to point out the fracture area more precisely to make the algorithm proper for education. The algorithm determined negative images will not present any heatmap to prevent confusion. The predicted fracture sites will be highlighted in positive images to guide the participants.

### Experimental protocol and randomization

**Fig. 1:**
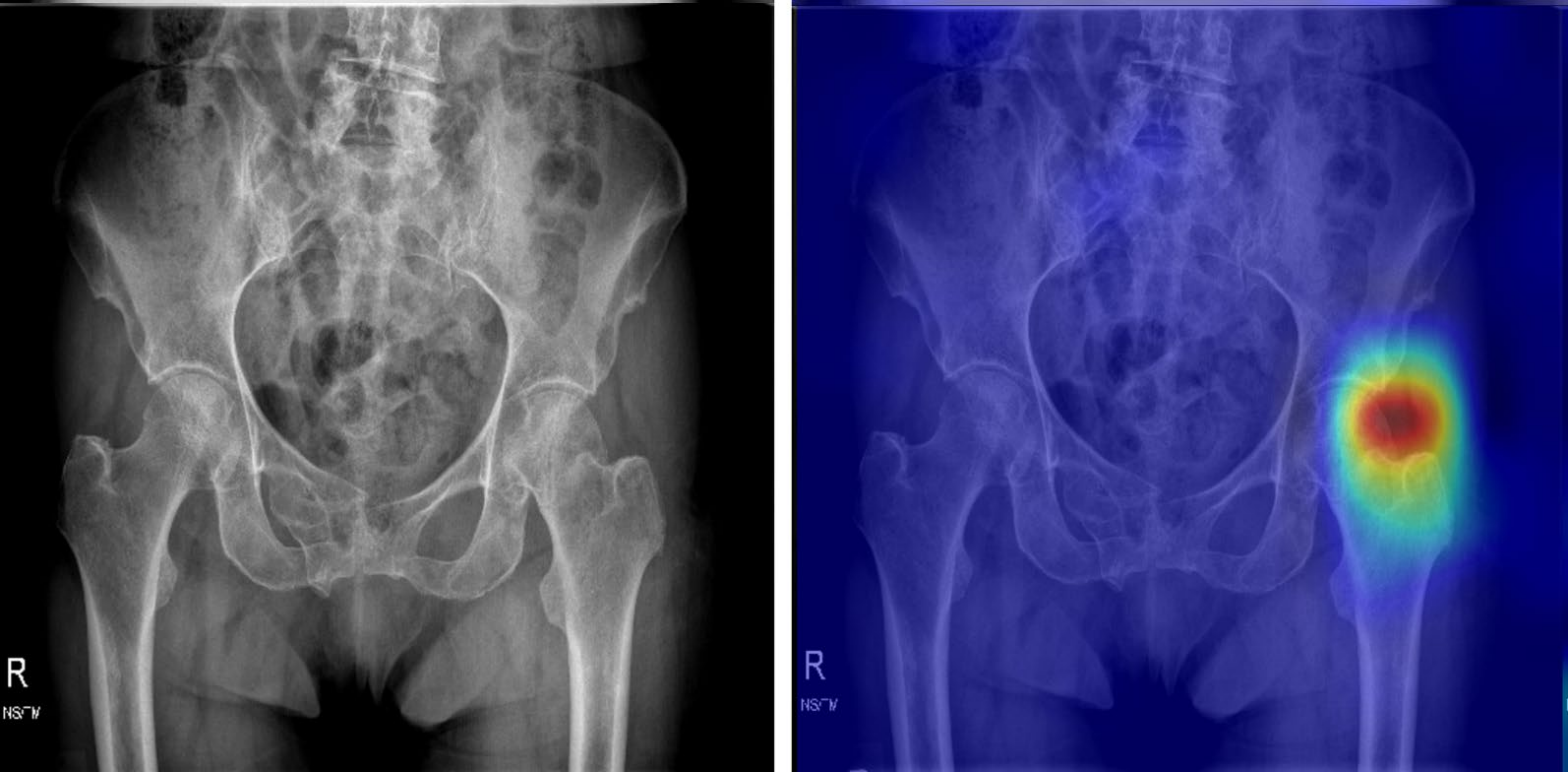
The demonstration of AI-augmented PXR with AI-introduced heatmap images. The augmented image includes the original plain pelvic film and one highlighted image that indicates the possible fracture area

**Fig. 2:**
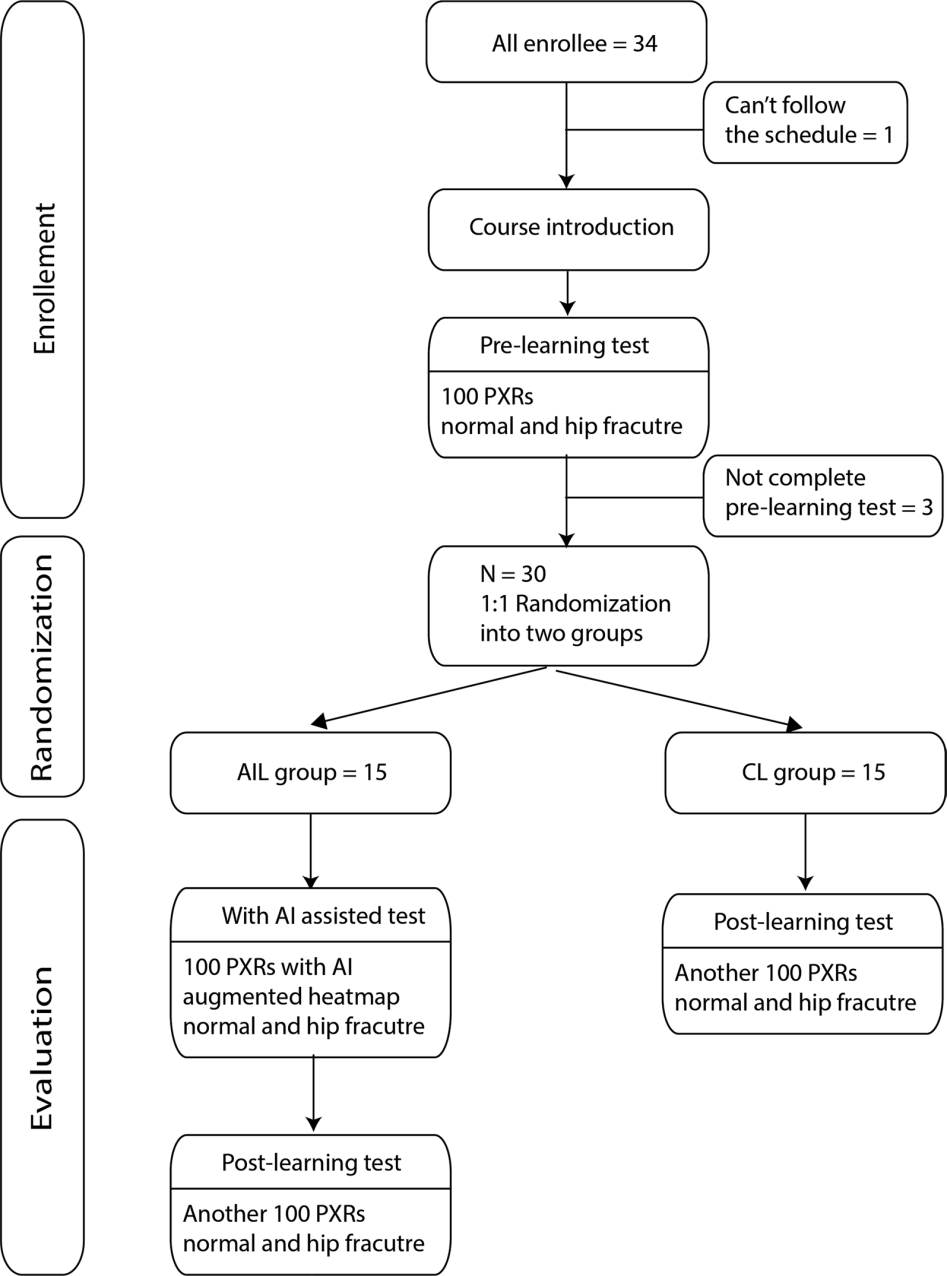
Diagram describing the randomized method of our study

The participants received a test composed of 100 randomized images from the 2017 PXR dataset, and the accuracy of the results was defined by the prelearning score. Furthermore, we randomized the students with the simple method of flipping a coin. They were divided into two groups: the AI-assisted learning (AIL) group and the conventional (CL) group. In the CL group, the students received a postlearning test composed of another 100 PXR images 2 weeks after the first test, and the performance was defined as the postlearning test score. In the AIL group, the students took one additional test composed of 100 AI-augmented PXRs with AI-introduced heatmap images (as shown in Fig. 1) 1 week after the prelearning test, and the performance was defined as the WithAI score. One week later, the AIL students took another test composed of 100 more PXR images without AI augmentation, and the performance was defined as the postlearning score. The study flow is shown in Fig. 2. To evaluate the improvement in this learning process, we defined the gained score as the postlearning score minus the prelearning score.

### Main outcome measures

The primary outcome of the study was the difference between the study groups in overall gained score. In addition, the gained score for the AIL group was calculated and analyzed to determine which group of students achieved the most improvement after AI-assisted training.

### Statistical analysis and software

The Shapiro–Wilk test was used to test whether the distribution was normal. Student’s t-test was used to compare the quantitative variables. Subsequently, the paired t-test was used to compare the accuracy scores to the corresponding baseline scores in each group. One-way analysis of variance (ANOVA) was used to compare the differences in improvement between the pre- and postlearning scores in the two groups. The Pearson correlation was used to compare the correlation between gained accuracy and prelearning accuracy. Statistical analyses were performed with SPSS v 20.0 for Macintosh (SPSS, Inc., Chicago, IL, USA). A *p* value < 0.05 was considered statistically significant.

## Results

**Fig. 3:**
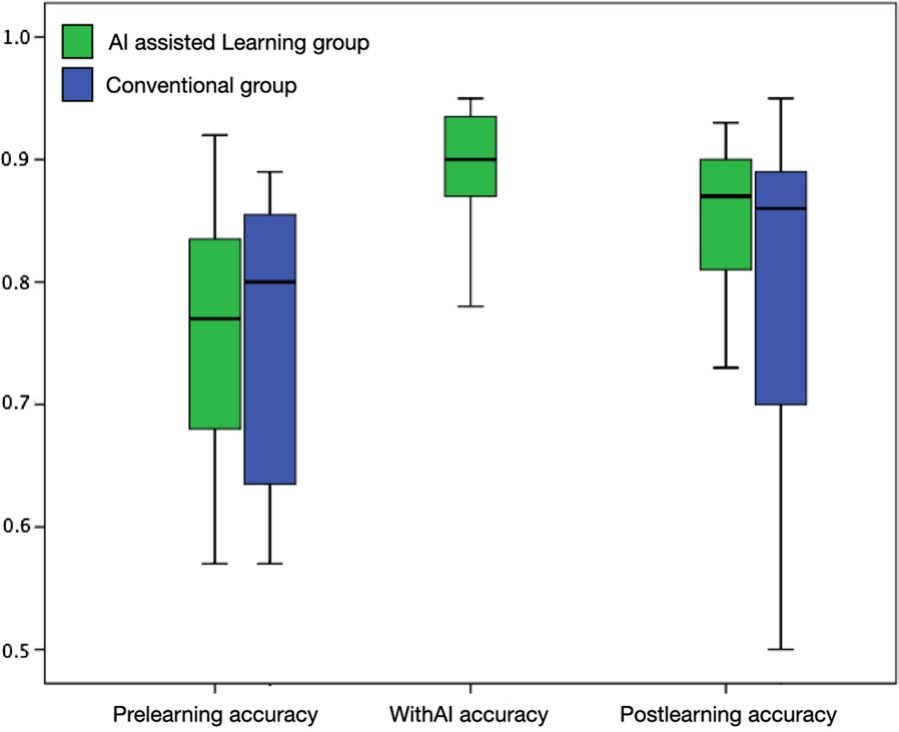
The box-plot graph of the diagnostic accuracy of the two groups

**Fig. 4:**
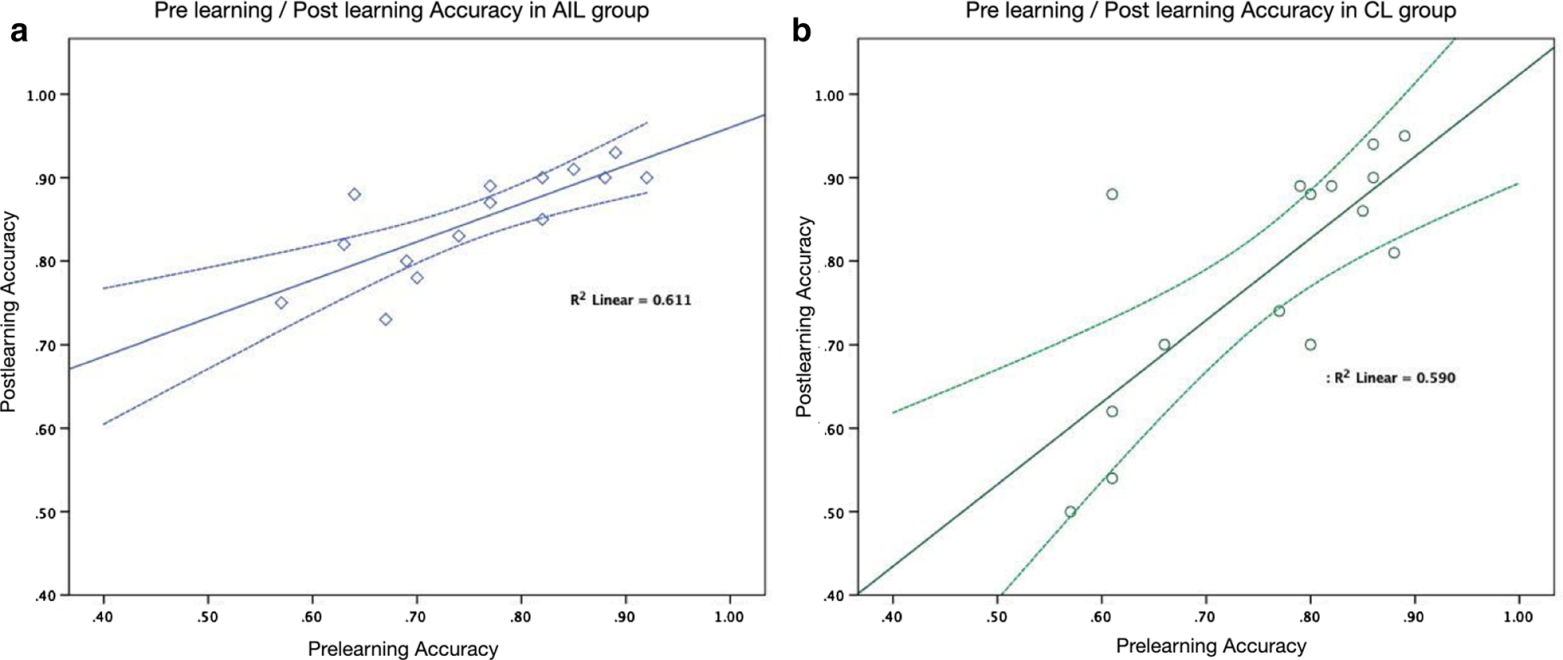
The prelearning/postlearning accuracy plot of the AI-assisted learning group and the conventional group

This study enrolled 34 medical students within the range of 22–32 years old who were randomly divided into the AIL and CL groups. Three of the students dropped out because they could not adhere to the evaluation schedule, and one could not complete the course. Therefore, a total of 30 students completed this study. The participants’ age, gender distribution, prelearning accuracy, sensitivity, and specificity were comparable in the two groups, as shown in Table 1. In the CL group, the postlearning score (78.66 ± 14.53) was higher than the prelearning score (75.86 ± 11.36), but there was no significant difference (*p* = 0.264). In the AIL group, all the participating students showed remarkable improvement with AI support. The WithAI score (88.87 ± 5.51) was significantly higher than the prelearning score (75.73 ± 10.58, *p* < 0.01). Moreover, the postlearning score (84.93 ± 14.53) was also better than the prelearning score (*p* < 0.01), as shown in Table 2 and Fig. 3. Figure 4 demonstrates the shift of the scores of both groups. In the AIL group, the prelearning/postlearning plot shows considerable improvement after AIL, as shown in Fig. 4a; however, the learning effect in the CL group was less significant, as shown in Fig. 4b.

**Table 1:** Comparison between AI-assisted learning and conventional groups

	AI-assisted learning group	Conventional group	*p* value
Case number	15	15	
Age (mean + SD)	24.00 ± 1.60	24.67 ± 3.13	0.471
Gender			
Male	9	10	0.740
Female	6	5	
Pre-learning accuracy	75.73 ± 10.58	75.86 ± 11.36	0.974
Pre-learning sensitivity	77.33 ± 18.15	72.53 ± 15.37	0.441
Pre-learning specificity	74.13 ± 17.42	79.20 ± 15.47	0.407
With AI accuracy	88.87 ± 5.51	–	
With AI sensitivity	94.93 ± 5.23	–	
With AI specificity	82.80 ± 10.50	–	
Post-learning accuracy	84.93 ± 14.53	78.66 ± 14.53	0.141
Post-learning sensitivity	86.40 ± 6.42	75.47 ± 22.10	0.084
Post-learning specificity	83.47 ± 14.03	81.87 ± 14.27	0.759

**Table 2:** Comparison of the gain scores of diagnostic performances between AI-assisted learning and conventional groups

	AI-assisted learning group	Conventional group	*p* value
Gained accuracy	9.2 ± 6.9	2.8 ± 9.3	**0.042**
Gained sensitivity	9.1 ± 1.7	2.9 ± 1.4	0.299
Gained specificity	9.3 ± 1.8	2.6 ± 1.1	0.237

Bold indicates *p* < 0.05 which was considered statistical significance

**Fig. 5:**
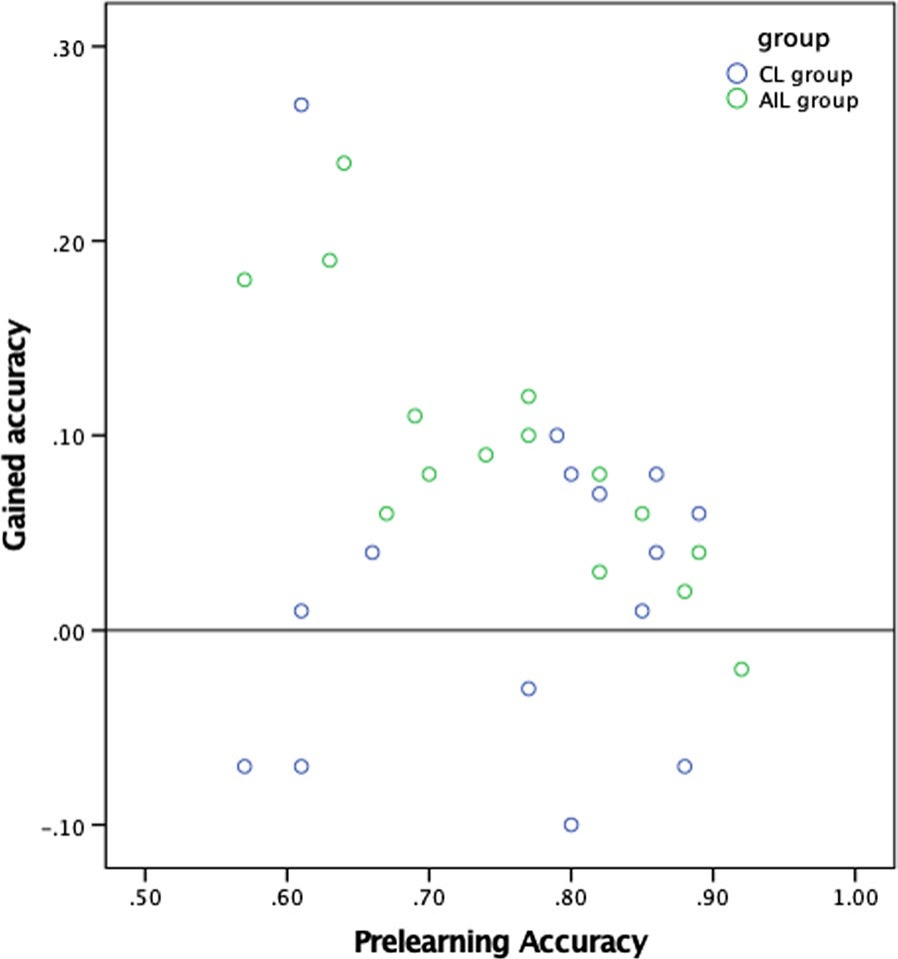
The comparison of gained accuracy with prelearning accuracy in the two groups

In comparing the AIL and CL groups, we identified a significantly higher gained accuracy in the AIL group (9.2 ± 6.9) than in the CL group (2.8 ± 9.3), *p* = 0.042, as shown in Table 3. As shown in Fig. 5, most of the AIL students obtained better scores and were located above the line of improvement. Just one participant’s score was below the line. However, in the CL group, five students (33%) did not improve and even had worse results after the learning.

**Table 3:** The learning efficiency and post-learning performance of AIL and CL groups

	Pre-learning	With AI	Post-learning
AIL group			
Accuracy	75.73 ± 10.58	88.87 ± 5.51*	84.93 ± 14.53^⍏^
		*Pre-learning versus with AI: ***p*** **<** **0.01**	^⍏^Pre-learning versus post-learning: ***p*** **<** **0.01**
CL group			
Accuracy	75.86 ± 11.36		78.66 ± 14.53^⍏^
			^⍏^Pre-learning versus post-learning: *p* = 0.264

Bold indicates *p* < 0.05 which was considered statistical significance

In the AIL group, the gained accuracy improvement was greater in the participants who had lower prelearning accuracy than in those who had higher prelearning accuracy. The strong inverse correlation is statistically significant, and the Pearson correlation coefficient (Pearson's *r*) is − 0.83 (*p* < 0.01). However, in the CL group, the gained accuracy showed no correlation with prelearning accuracy (Pearson's *r*: − 0.02, *p* = 0.94), as presented in Table 4.

**Table 4:** The correlation between gained accuracy with pre-learning accuracy in AI-assisted learning group and conventional learning group

	Pre-learning accuracy	
	*r*	*p* value
AIL group		
Gained accuracy	− 0.83	**< .01**
CL group		
Gained accuracy	− 0.02	.94

Bold indicates *p* < 0.05 which was considered statistical significance

## Discussion

In this study, we presented AI-augmented medical education that can help medical students efficiently learn to detect fracture of the hip; the gained accuracy score improved with a significant difference between the two groups. This is the first study to integrate AI into medical image education. In the AIL group, the WithAI score was significantly higher than the prelearning score. The AIL students who had been coached by the HipGuide system, even without AI-augmented image support, understood the key features of fracture and had learned how to identify the correct diagnosis. The postlearning score in the AIL group was significantly better than the prelearning score. Thus, the AI-based education system demonstrated its utility in improving trainee performance through quality measures that should be integral to the improvement in medical education, especially with the utilization of AI [7, 17, 18]. Furthermore, we found that the HipGuide system particularly helped novice students who had lower prelearning scores. The gained scores showed a strongly inverse correlation with the prelearning scores, which indicates that HipGuide helped novice students more than it helped their experienced classmates.

In the past, medical image training has relied on the traditional apprenticeship model. Because this model depends on trainee relationships, and because there is limited time available to review preliminary reports with a staff radiologist, gains in knowledge and skills can vary among trainees [19–21]. Hence, this apprenticeship education model is characterized by ever-increasing workload demands on both attending physicians and trainees and can be improved by a better understanding of relations between humans and tools. [8, 22, 23]. In conventional image teaching, students are expected to first learn diagnostic characteristics from educators and practice afterward. AI in medical education is still in the development stage [2, 7, 24–26]. AI can empower flipped classroom-like teaching [27, 28] and complete the existing bottom-up platforms used to teach radiology [29]. It has been argued that case-based learning should be implemented because it is more effective than traditional top-down approaches and is preferred by radiology educators and students [30, 31]. In the hip fracture detection task, the medical students had fundamental knowledge of anatomy, pathology, and the principle of imaging. The heatmap generated by AI provided an opportunity for the students to connect this domain knowledge and generate a new understanding of radiographic interpretation skills. Thus, they could improve their diagnostic accuracy with limited learning cases. Visualization techniques such as grad-CAM provide a rationale for humans to understand the black box mechanism [32]. Students can utilize AI to supplement unsupervised learning during personal time. AI can be used for low-level supervision, while attending and staff physicians can continue to provide high-level supervision [14, 33]. This change would allow human educators to tailor training methods and lesson content to their students’ strengths and weaknesses, promoting bidirectional information exchange and saving training time. Thus, AI supports unique education platforms that balance a standardized curriculum with inherently individualized learning.

A well-designed self-learning platform with AI augmentation will be an adequate model for medical education in the next generation. Kolachalama and Garg proposed the integration of machine learning-related content in medical education. They recommended integrating real-world clinical examples into machine learning courses and applying practical guidelines to choose the best tools [34, 35]. Because of a current lack of tutors’ direct access to appropriate AI education, AI-assisted teaching methods are rarely embedded in undergraduate and graduate medical education training. Creating a user-friendly automated platform to help both trainers and trainees is essential for developing an AI educational system [36]. In this study, we presented a straightforward pathway to support AI-based technology that can also help medical students or novice doctors learn and obtain experience quickly. This study offered a method to improve medical students’ learning of medical images with instinctive AI diagnostic support.

## Limitations

There have been significant breakthroughs in AI in medical diagnostics in recent years. Our study provides evidence supporting the proposal that AI can help medical education. There are still some limitations. There have been few, if any, direct comparisons between conventional and AI-augmented medical image education. To determine and verify the utility of AI in precision education, trainee performance must be assessed through reliable and valid measures [7]. Second, the sample size was relatively small (*N* = 30), which might affect the power of the study, although an analysis of 560 studies indicated that this number is within the normal range of experimental groups [37]. Third, because the educator curates and collects the data for AI algorithm development, selection bias might exist due to the data distribution. Fourth, given our training capacity, we decided to recruit thirty-six participants for this study and opened it to residents on a first-come, first-served basis. There might be selection bias because we assume that those who chose to participate had high motivation and interest in learning novel technology. Further, when we proceeded with this study, all the medical students also had access to other approaches for learning medical image diagnosis. Because all the medical students may not have had equal learning opportunities, there may have been unpreventable bias. Finally, owing to the limitations of the technology, we can offer only the heatmap rather than using an arrowhead to point directly to a lesion, which is less user-friendly. However, despite the limitations of this study, we demonstrated that AI can shorten the learning curve in learning to read PXRs.

## Conclusion

In this study, we demonstrate that AI is viable for augmenting medical education and can shorten the learning curve in learning to read PXRs. With AI assistance, students can learn to efficiently read medical images. In addition, AI-based training can elevate diagnostic accuracy even further for students whose performance is initially poor. The next generation of students may learn how AI technologies are used in practice, and similar tools will also be used to enrich their education.

## Data Availability

The authors declare the data that support the findings of this study are available on request from the corresponding author C.T. Cheng.

## Abbreviations

AI: Artificial intelligence
AIL: AI-assisted learning
ANOVA: Analysis of variance
CL: Conventional learning
PACS: Picture archiving and communication system
PXRs: Pelvis AP view radiographs
